# Morphology of the Myocardium after Experimental Bone Tissue Trauma and the Use of Extracellular Vesicles Derived from Mesenchymal Multipotent Stromal Cells

**DOI:** 10.3390/jpm11111206

**Published:** 2021-11-15

**Authors:** Igor Maiborodin, Marina Klinnikova, Sergey Kuzkin, Vitalina Maiborodina, Sergey Krasil’nikov, Aleksandra Pichigina, Elena Lushnikova

**Affiliations:** Institute of Molecular Pathology and Pathomorphology, Federal State Budget Scientific Institution “Federal Research Center of Fundamental and Translational Medicine” of the Ministry of Science and Higher Education of the Russian Federation, Timakova St., 2, 630117 Novosibirsk, Russia; margen@ngs.ru (M.K.); acutus@mail.ru (S.K.); mai_@mail.ru (V.M.); professorkrasilnikov@rambler.ru (S.K.); apichigina@yandex.ru (A.P.); pathol@inbox.ru (E.L.)

**Keywords:** multipotent stromal cell extracellular vesicles, myocardium, bone tissue defect of a limb, edema, hemorrhages, leukocyte infiltration, sclerosis

## Abstract

The effect of extracellular vesicles (EVs) of various origins on the heart structures in the time of health and disease has been well studied. At the same time, data on the distribution of EVs throughout the body after introduction into the tissues and the possibility of the influence of these EVs on organs distant from the injection site are practically absent. It is also necessary to note a certain inconsistency in the results of various researchers: from articles on the direct absorption of EVs derived from mesenchymal multipotent stromal cells (MSC EVs) by cardiomyocytes to the data that the heart is inherently immune to drug delivery mediated by nanoparticles. In this regard, the morphological changes in the myocardium of outbred rabbits of both sexes weighing 3–4 kg were studied at various times after experimental trauma of the bone tissue in the proximal condyle of the tibia (PCT) and the use of MSC EVs. As a result of modeling the PCT defect, rabbits develop myocardial edema in the heart muscle by the 3rd day, their lymphatic vessels expand, and then, on the 7th day, the blood vessels become dilated. In the myocardium, the relative and absolute contents of neutrophils, erythrocytes, and macrophages increase, but the percentage of lymphocytes decreases. By day 10, almost all of these changes return to their initial values. The detected transformations of the myocardium are most likely due to the ingress of detritus with the blood flow from the PCT. The use of MSC EVs to influence the regeneration of damaged tissue of PCT promotes earlier dilatation of the blood vessels of the heart with pronounced diapedesis of erythrocytes or even hemorrhages, prolongation of edema, the formation of blood clots in vessels with obliteration of their lumen, sclerotic transformation of vascular walls and paravascular tissues. In the myocardium, the number density of neutrophils, the percentage of lymphocytes, and neutrophils become smaller, with a simultaneous increase in the relative numbers of erythrocytes and macrophages, and changes in the content of macrophages remained until the end of the observation—up to 10 days after the surgery. The discovered effect of MSC EVs is most likely associated with the suppression of the activity of the inflammatory process in the PCT area, which, in turn, was caused by a longer ingress of detritus with blood flow into the myocardium. The absence of statistically significant differences between changes in the myocardium of the left and right ventricles may indicate that both detritus from the surgical site and MSC EVs affect the heart spreading through the coronary artery system.

## 1. Introduction

Mesenchymal multipotent stromal cells (MSCs) are cellular instruments with good potential for regenerative medicine due to their strong immunosuppressive and reparative abilities. However, after injection, MSCs remain in the tissues for a very short time [1,2,3,4,5]. This indicates that the prolonged action of MSCs may be mediated by their paracrine effects, such as exosomes/extracellular vesicles (EVs). Exosomes are now considered as a key regenerative component produced by MSCs and progenitor cells. MSC EVs are rapidly absorbed by native cells and are ideal natural transporters for the exchange of contents between cells, as they carry many signaling biomolecules, including microRNAs, proteins, enzymes, cell surface receptors, growth factors, cytokines, and lipids, which can modulate the state and function of cell targets. MSC EVs exhibit cardioprotective, immunomodulatory, and reparative abilities, suppress inflammation and apoptosis, stimulate angiogenesis, and enhance the proliferation and differentiation of resident tissue MSCs, including cardiac ones [6,7,8].

The literature contains a large number of publications devoted to the influence of exosomes of various origins on the heart structures in the time of health and disease. At the same time, data on the distribution of EVs throughout the body after different routes of administration are practically absent: found only the post of Z. Wan et al. [9] and the opinion of S. Femminò et al. [8] that circulating EVs can target cardiovascular structures, and EVs derived from MSC of bone marrow origin can be transmitted to distant tissues, including the myocardium, via blood flow after local injection into distant organs and tissues [10]. It is also necessary to note a certain inconsistency in the results of various researchers: from articles on the direct absorption of MSC EVs by cardiomyocytes [11] to the data that the heart is inherently immune to drug delivery mediated by nanoparticles [12].

In connection with the above, the morphological changes in the myocardium were studied after experimental bone tissue injury and the use of MSC EVs.

## 2. Materials and Methods

This research is based on findings obtained when studying the samples of the heart of outbred rabbits of both sexes weighing 3–4 kg at various times after the injection of MSC EVs into an artificially created defect of the proximal condyle of the tibia (PCT) with subsequent installation of screw titanium implants. The manipulations did not cause pain to animals and were carried out in compliance with Russian legislation: GOST 33215-2014 (Guidelines for accommodation and care of laboratory animals. Rules for the equipment of premises and organization of procedures) and GOST 33216-2014 (Guidelines for accommodation and care of laboratory animals. Rules for the accommodation and care of laboratory rodents and rabbits). Work approved by the committee for biomedical ethics (20 November 2020, decision № 32) of a Federal State Budget Scientific Institution “Federal Research Center of Fundamental and Translational Medicine” (FRC FTM).

### 2.1. Preparation, Cultivation, and Characteristics of MSCs, Isolation of MSC EVs

Due to the numerous literature publications that indicate undifferentiated MSCs do not have surface antigens that determine their foreignness and do not initiate an immune system response even after allogeneic and xenogenic transplantation, and that the inflammation and proliferation of allogeneic T-lymphocytes are also suppressed, it is possible to use MSCs isolated from some animals for implantation in other individuals, even other species [13,14]. Similar data were obtained in the study of the immunogenicity of EVs derived from human umbilical cord MSCs. EVs were given to rabbits, guinea pigs, and rats, and EVs have been found to have a protective effect on weight loss and had no adverse effects on liver or renal function. Other detections, such as hemolysis, vascular and muscle stimulation, systemic anaphylaxis, and pyrogen and hematology indexes, also showed exosomes were applicable. Thus, EVs from human umbilical cord MSCs are well tolerated in animal models [15]. Moreover, there is evidence of the possibility of using EVs of plant origin for the treatment of various pathological processes in mammals. Currently, there is evidence that EVs of plant origin may be involved in plant–cell communication as well as in interspecies communication between plants and animals. For example, a plant-derived miRNA such as miR-168 has been reported to enter the circulation of rice-fed mice enclosed in EVs and to modulate the expression of target genes [16]. EVs are released from microorganisms and may participate in interspecies communication in the gut [17]. Based on the above, a decision was made on the possibility of transplanting EVs obtained from rat MSCs into rabbits.

MSCs were obtained from the bone marrow of a male Wag inbred line rat weighing 180 g and aged 6 months and then were characterized and cultured as described in our previous studies [2,3,5,10,18]. Isolated MSCs express some characteristic MSC markers (CD73, CD90, and CD105) and do not express the hematopoietic markers (CD14, CD20, CD45, and CD34). The culture nutrient medium containing fetal bovine serum was used for MSC growth and preliminarily subjected to complete gradient centrifugation to purify it from its own EVs. At the stage of stationary growth of a stable culture of the 3rd MSC passage, when the confluence of the cell monolayer reached 80–90%, a conditioned medium was collected, from which MSC EVs were isolated as recommended in the literature [10,18,19]. To remove cells, cell debris, apoptotic bodies, and large vesicles, the conditioned medium was centrifuged sequentially: 10 min in case of 300 g, 10 min in case of 2000 g, and 30 min in case of 12,000 g. EVs were precipitated by centrifuging the supernatant for 2 h in case of 100,000× *g* and resuspended in saline with phosphate buffer. MSC EVs were analyzed by electron transmission microscopy and flow cytometry. Relevant marker for EVs secreting MSCs, tetraspanins CD9, CD63, and CD81 were detectable [20,21].

When preparing samples for research, the objects were sorbed onto a copper grid covered with a formvar film for 1 min and contrasted with a 2% phosphotungstic acid solution for 10 s. The grids were studied in the transmission mode of a Jem1400 electron microscope (Jeol, Japan); images were obtained using a Veleta digital camera (Olympus Corporation, Japan). Particle size was determined in 5–8 randomly selected fields of view at 60,000 times magnification using the iTEM software package (Olympus Corporation, Japan). More than 90% of the objects had a diameter of 70–90 microns and a three-layer membrane. The isolated extracellular particles were adsorbed onto 4 μm aldehyde/sulfate latex particles (Invitrogen, 37304) [22] and analyzed on a NovoCyte™ cytometer using its software (ACEA Biosciences Inc., Santa Clara, CA, USA). To detect EVs, antibodies specific to marker-specific exosome proteins (Bio-Rad, MCA4754F, FITC mouse IgG1, k) were used. An isotype control (Bio-Rad, MCA1209, isotype control, FITC mouse IgG1, k) was used to measure nonspecific sorption. In each experiment, no less than 30,000 events were counted. The amount of MSC EVs was determined by the protein content in the precipitate using a commercial Qubit protein assay kit (Thermo Fisher Scientific, Kalamazoo, MA, USA) and a Qubit^®^ 3.0 fluorometer (Thermo Fisher Scientific, Kalamazoo, MA, USA).

### 2.2. Introduction of MSC EVs into a Bone Defect

Surgical intervention was performed in compliance with all the rules of asepsis and antiseptics under general intravenous anesthesia with propofol. In both PCT of rabbits, standardized 4 mm holes were created with a 2 mm dental bur cooled by sterile saline solution [10,18].

Next, an insulin syringe was used to fill the bone defect with physiological saline prepared in phosphate buffer (pH = 7.3) [23] (control, 9 rabbits), or 19.2 μg of MSC EVs in saline solution was injected for each limb (experiment, 10 animals). The MSC EVs dose was selected based on the average dose recommended by other researchers: 10–20 μg/mL [24]; 0.6 μg, 5 μg, and 50 μg [25]; 50 μg for the same bone tissue defect of the PCT [10,18]; 100 μg immediately after surgery and weekly for 12 weeks [23]. After 10–20 s, titanium screw implants (catalog number IS 358; 3.5 × 8 mm with a rough surface; 3S, Israel) were inserted with a stable primary fixation up to 30 Ncm, and the surgical wound was sutured layer by layer without tension [10,18].

After 3, 7, and 10 days, the animals were sacrificed by dislocation of the cervical vertebrae. Each group consisted of 3–4 animals, 19 animals in total.

### 2.3. Morphological Research Methods

A fragment with a thickness of about 5 mm was cut from the hearts of rabbits so that the sample contained the right and left atria and ventricles with the maximum area of the cavities. Then, the right and left parts were divided along the septa and processed separately. Samples of the myocardium were fixed in 4% paraformaldehyde solution in phosphate buffer (pH 7.4) for at least 1 day, then dehydrated in the Isoprep reagent (Biovitrum, St. Petersburg, Russia), clarified in xylene, and embedded in a Thermo Scientific™Histoplast Paraffin (Richard-Allan Scientific, Subsidiary of Thermo Fisher Scientific, Kalamazoo, MI, USA). The heart was sampled in sections with a thickness of 5–7 μm, which were stained with hematoxylin and eosin. To assess the number and distribution of lymphocytes and macrophages on the sections, an indirect immunoperoxidase reaction with monoclonal antibodies against the CD5 or CD68 antigen (Dako, Denmark), respectively, was carried out. Determination of neutrophils and erythrocytes on the sections was made based on the visual characteristics. Lymphatic vessels were distinguished from blood vessels in accordance with the recommendations of Head and Seeling [26]. The sections were studied under a light microscope Axioimager M1 (Zeiss, Germany) at magnification up to 1200 times.

To obtain the necessary morphometric data, the images were measured using the digital video camera of this microscope with the help of the Axiovision morphometry software package (Zeiss, Germany). The vascularization of the right and left myocardium was determined on random sections. To determine the severity of inflammatory infiltration (lymphocytes, neutrophils, macrophages, and erythrocytes), the images obtained with a digital video camera of a microscope were measured on a computer screen using the software of the Axiovision morphological module (Zeiss, Germany). When using a lens with a magnification of × 40, the area of the rectangular image was 8.7 × 10^4^ μm (sides 350 × 250 μm). Three to five measurements of different areas were performed on each sample according to the instructions that it is enough to study 3 sections from each object to obtain statistically significant results [26].

During statistical processing of the obtained data, the arithmetic mean and standard deviation were determined. The significance of the difference between the compared mean values was determined on the basis of Student’s *t*-test; the difference between the compared series with the confidence level of 95% and higher was considered significant. The calculations took into account that the distribution of the studied characters was close to normal [27].

## 3. Research Findings and Their Discussion

It should be noted that previously, we have published the features of the regeneration of the PCT defect after of MSC EVs application. The screw dental implants were installed in the proximal condyles of the tibia of outbred rabbits without and with the preliminary introduction of 19.2 μg MSC EVs into each bone tissue defect. In 3, 7, and 10 days after the operation, the density of bone tissue adjacent to different parts of the implant using an X-ray unit with a densitometer was measured. In addition, the histological examinations of the bone site with the hole from the removed device and the soft tissues from the surface of the proximal tibial condyle in the area of intrabone implants were made. It was found that 3 days after implantation with the use of MSC EVs, the bone density was statistically significantly higher by 47.2% than after the same implantation but without the injection of MSC EVs. It is possible that as a result of the immunomodulatory action of MSC EVs, the activity of inflammation decreases, and, respectively, the degree of vasodilation in bones and leukocyte infiltration of the soft tissues are lower, in comparison with the surgery performed in the control group. The bone fragments formed during implantation are mainly consolidated with each other and with the regenerating bone. Day 10 demonstrated that all animals with the use of MSC EVs had an almost complete fusion of the screw device with the bone tissue, while after the operation without the application of MSC EVs, the heterogeneous histologic pattern was observed, from almost complete osseointegration of the implant to the absolute absence of contact between the foreign body and the newly formed bone. Therefore, the use of MSC EVs during the introduction of dental implants into the proximal condyle of the tibia of rabbits contributes to an increase in the bone tissue density near the device after 3 days and to the achievement of consistently successful osseointegration of implants 10 days after the surgery [28].

Earlier, we proved the possibility of entering MSC EVs into the myocardium of rabbits after injection into the damaged PCT. When administered intravenously, MSC EVs immediately pass through the lungs along with the blood and regularly spread to all organs. When administered intraperitoneally, they are absorbed either into the blood or into the lymph and are quickly disseminated throughout the body. The possibility of generalized spread of MSC EVs to distant organs in case of local intratissular administration remains unexplored. However, it is impossible to exclude MSC EV influence on tissues distant from the injection site due to the active or passive migration of these injected nanoparticles through the vessels. The previous research was based on findings obtained when studying the samples of lungs, heart, spleen, and liver of outbred rabbits at various times after the injection of EVs derived from MSCs of bone marrow origin and labeled by PKH26 into an artificially created defect of the PCT. After the introduction of MSC EVs into the damaged proximal condyle of the tibia of rabbits, these MSC EVs can be found most frequently in the lungs, myocardium, liver, and spleen. MSC EVs enter all of these organs with the blood flow. The lungs contained the maximum number of labeled MSC EVs; moreover, they were often associated with detritus and were located in the lumen of the alveoli. In the capillary network of various organs except for the myocardium, MSC EVs are adsorbed by paravasal phagocytes; in some cases, specifically labeled small dust-like objects can be detected throughout the entire experiment—up to 10 days of observation [10].

**Figure 1:**
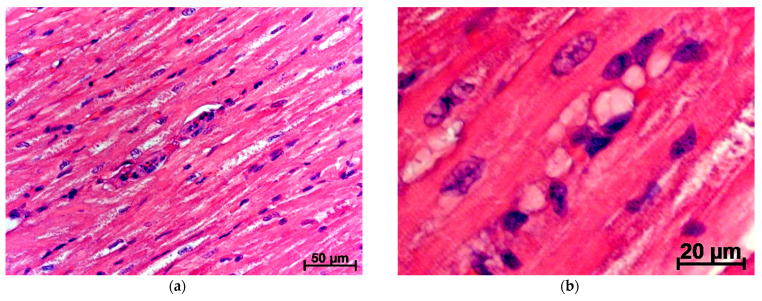

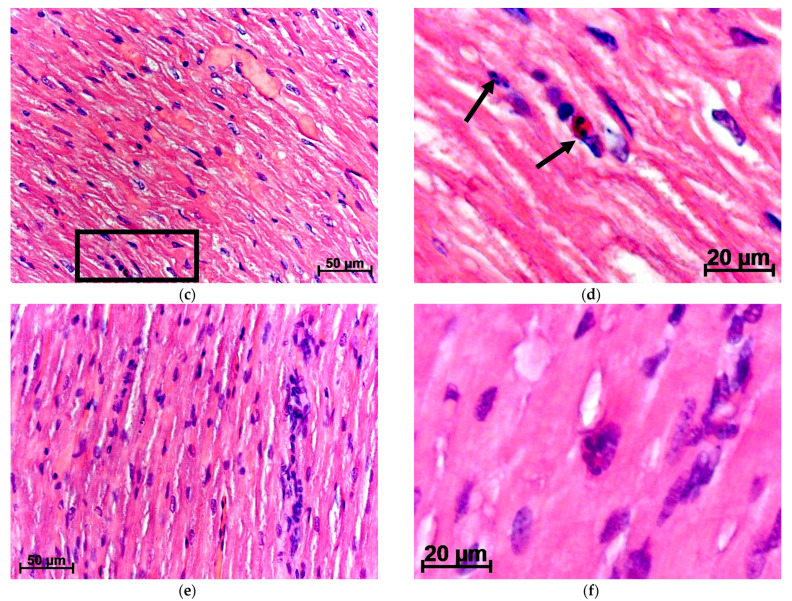
Changes in the myocardium of rabbits after damage of PCT. Stained with hematoxylin and eosin: (**a**,**b**) 3 days; (**c**,**d**) 7 days; (**e**,**f**) 10 days after surgery; (**a**) increased leukocyte infiltration; (**b**) dystrophic changes in cardiomyocytes, accumulation of a significant volume of edematous fluid between them; (**c**) hyperemia, dilation of blood vessels and diffuse leukocyte infiltration, edema has become smaller, but still quite pronounced; (**d**) neutrophils (arrows) in the myocardium; (**e**) edema persists but hyperemia and lymphostasis normalized; (**f**) a large number of macrophages.

Three days after only damage to the PCT, a very pronounced expansion of the interstitial spaces and lymphatic vessels was found in the myocardium of the left ventricle: 15.4 and 4.3 times, respectively, relative to the intact control. At the same time, the blood vessels remained undiluted. Diffuse and perivascular leukocyte infiltration was observed (Table 1) (Figure 1a,b).

During extensive surgical operations with damage to large tissue arrays, biologically active substances, tissue, and cellular detritus enter the blood flow and spread throughout the body, including into the heart muscle. Debris and biologically active compounds, once in the myocardium, can affect cardiomyocytes and the vessels, mainly lymphatic ones. The myocardial lymphatic vessels and their initial parts—interstitial (intercellular) spaces are blocked and expanded.

The percentage of lymphocytes among leukocytes on day 3 was statistically significantly less by 25.9%, but the relative and absolute content of neutrophils became 4.1 and 7.4 times higher. The number of macrophages per unit area of the tissue section after 3 days increased by 96.1%, compared with intact control (Table 1) (Figure 1a,b). From immunocompetent cells in places of appearance or increase in the concentration of antigenic substances, neutrophils appear first. Most likely, this is the basic reason for the high number of these leukocytes in the myocardium after PCT damage, i.e., due to a sharp increase in the percentage of neutrophils, the relative number of lymphocytes decreases.

The percentage and numerical density of red blood cells in myocardial tissue on the 3rd day after the creation of the PCT defect were 2.4 and 4.1 times, respectively, greater than in intact animals (Table 1). Antigenic and toxic substances entering the myocardium from PCT with a defect trigger the migration of leukocytes, but they also disturb blood microcirculation. It is most likely that the increase in the content of extravascular erythrocytes in the heart muscle is due to violations of the permeability of blood capillaries under the influence of biologically active substances coming via the blood flow from PCT.

**Figure 2:**
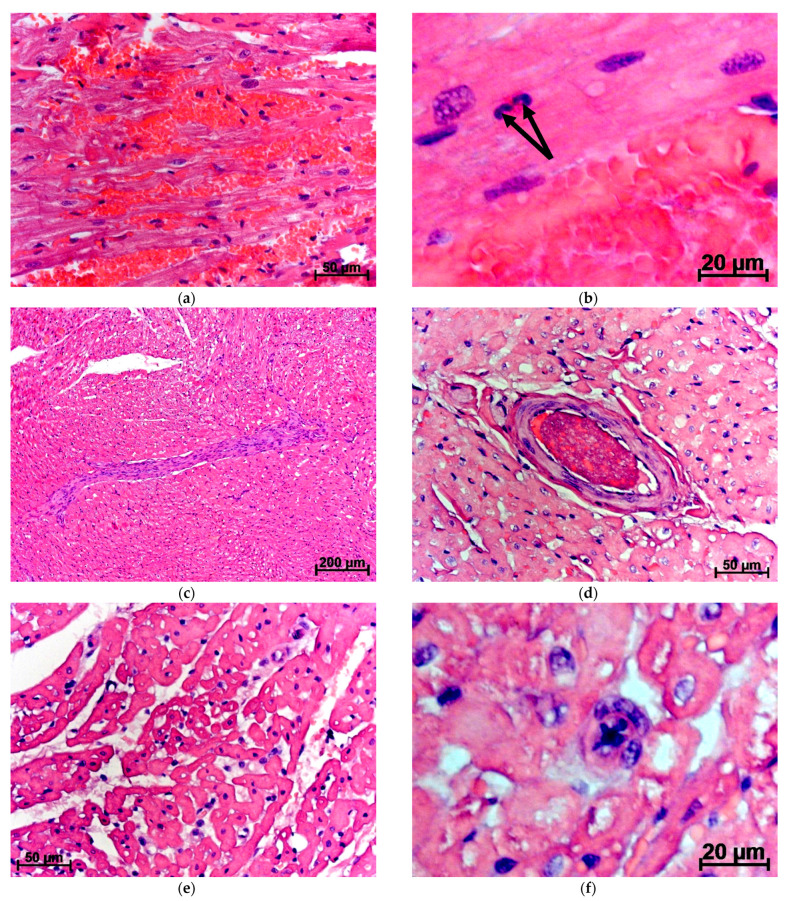
Changes in the rabbit myocardium after PCT damage with the subsequent introduction of MSC EVs. Stained with hematoxylin and eosin: (**a**,**b**) 3 days; (**c**,**d**) 7 days; (**e**,**f**) 10 days after surgical intervention: (**a**) severe hyperemia with diapedesis of erythrocytes and hemorrhages; (**b**) pronounced hyperemia, neutrophils (arrows) in the myocardium; (**c**) connective tissue layer and swelling of the heart muscle; (**d**) swelling of the heart muscle, fibrinoid impregnation of the walls and thrombosis of a large artery; (**e**) severe edema and lymphostasis, high level of leukocyte infiltration; (**f**) vasculitis with obliteration of the vascular lumen in the edematous heart muscle.

Three days after the PCT damage with the subsequent introduction of MSC EVs, in the myocardium of the left ventricle, even more pronounced edema was observed; this already occurred, together with a very significant expansion of blood capillaries. In some cases, heart tissue, due to hyperemia and even thrombosis, was imbibiated by red blood cells. On myocardial sections, the volume of blood and lymphatic vessels was 3.8 times and 78.6% larger, respectively, but the area of interstitial spaces became smaller by 46.9%, compared with after the same surgical intervention but without MSC EVs. In addition, the percentage of lymphocytes, neutrophils, and numerical density of neutrophils were statistically significantly lower by 26.3%; 80.5%, and 2.2 times, respectively. At the same time, the relative number of red blood cells and macrophages increased by 72% and 92%, respectively (Table 1) (Figure 2a,b).

According to the literature, MSC EVs suppress the proliferation and functional activity of cells of the lymphocytic and myeloid series [29,30,31]; thus, a decrease in the number of lymphocytes and neutrophils in the myocardium is possible. As a result of the immunomodulatory action of MSC EVs [32,33,34] in the site of application, in the PCT, the activity of the inflammatory process is suppressed. Detrite is not lyzed by leukocytes, migration, proliferation, and functional activities of which are suppressed by MSC EVs [29,30,31], and debris is not diluted and not eliminated outward through a surgical incision between the sutures but remains in the tissues [18], gradually absorbed into the blood and lymphatic vessels and spreading throughout the body ingress, including the heart. In the myocardium, when exposed to detritus from PCT, the same violations of microcirculation and lymph flow occur, as well as directly in the damaged peripheral tissues themselves. It is possible that a more pronounced expansion of the myocardial vascular components after the creation of a PCT defect with the introduction of MSC EVs leads to a slight decrease in the percentage of the area of interstitial spaces on the section. It is also likely that a significant dilatation of lymphatic vessels contributes to the accumulation of liquid contents of intercellular spaces there, leading to a slight decrease in their volume.

It cannot be ruled out that, due to the immunomodulatory effect of MSC EVs [32,33,34], toxins and antigens directly entering the heart, where they were transported with blood flow from tissues damaged during surgery, are not inactivated or inactivated more slowly and cause a more pronounced expansion or even blockade of both the microcirculatory part of the bloodstream and the initial parts of lymphatic pathways.

Since MSC EVs contain a high concentration of VEGF or trigger the synthesis of this cytokine by endothelial cells [35,36,37], it is possible that angiogenesis begins at the site of the presence of these EVs, and as a result of intensive contractile work of the myocardium, red blood cells leave young newly formed vessels with unformed walls via diapedesis pathway. In addition, the ectosomal fraction of EVs binds well to annexin V and can interact with prothrombin and blood clotting factor X for the formation of a prothrombinase complex and trigger blood coagulation [38,39]. As a result, thrombosis of part of the capillary network of the heart muscle is possible, where MSC EVs settle [10], with the expansion of arterioles and increased edema due to the release of the liquid part of the blood into the tissues.

By 7 days after the creation of the PCT defect, interstitial edema and expansion of lymphatic vessels in the rabbit hearts persisted. These changes were accompanied by the dilation of blood vessels. Volumetric densities of blood, lymphatic vessels, and interstitial spaces on myocardial sections of the left ventricle 7 days after surgery were higher in 6.1; 6.3, and 16.7 times, respectively, compared with intact control. The percentage of lymphocytes among leukocytes on the 7th day after surgery was lower by 48.4%, but the relative content of neutrophils and macrophages was higher by 4.1 and 2.1 times, respectively. The numerical densities of neutrophils and macrophages increased by 6.7 and 3.3 times, and the relative and absolute numbers of erythrocytes increased by 2.3 and 3.6 times, respectively (Table 1) (Figure 1c,d). Both neutrophils and the macrophage system react to an increase in the concentration of antigenic substances in the myocardium. First, in the heart muscle, the content of neutrophilic leukocytes increases, and then, after 7 days, the number of macrophages also grows.

After the creation of the PCT defect and the introduction of MSC EVs, it is necessary to note the presence in the heart muscle of all animals of rough connective tissue layers, perivascular sclerosis, thrombosis, and obliteration of the lumen in some vessels (Figure 2c,d). The formation of connective tissue layers may be associated with thrombosis and diapedesis of erythrocytes through the vascular wall of capillaries detected at the last term. Apparently, blood clots after surgery with using MSC EVs are formed in the vessels, where the ectosomal fraction of EVs penetrates [10], which can be a trigger for blood coagulation [38,39]. Thrombosed vessels become empty, their lumen is obliterated, and the walls are sclerosed. Gradually, instead of such vessels, connective tissue strands are formed.

Then, 10 days after the creation of the PCT defect, the myocardial edema in the left ventricle becomes smaller, and the size of the blood and lymphatic vessels normalizes but the leukocyte infiltration remains. Lymphocytes among leukocytes were less by 38.4% than at intact animals. At the same time, the relative and absolute contents of neutrophils after 10 days remained 2.1 and 3.6 times higher, and the percentage and numerical density of macrophages are 2.3 and 3.9 times greater, respectively, compared with intact control (Table 1) (Figure 1e,f). In other words, with the regeneration of damaged tissues of the hind limbs and the subsidence of inflammation, the influx of detritus into the blood and the volume of its migration into the heart decrease, the condition of the myocardium gradually normalizes: the severity of neutrophilic infiltration of heart tissues decreases with a parallel increase in the percentage of lymphocytes.

In addition, 10 days after the creation of the PCT defect using MSC EVs, in the heart muscle, the severity of edema and leukocyte infiltration remained at the same level. Obliteration of the vascular lumen and the presence of connective tissue layers along the course of some vessels were also preserved. The percentage and numerical density of neutrophils were 86% and 2.1 times, respectively, higher than after only damage to PCT (Table 1) (Figure 2e,f). It is possible that the immunomodulatory effect of MSC EVs [32,33,34] leads to a slowdown in the cleansing of tissues in the area of damage and, accordingly, prolongation of the ingress of detritus into the blood flow, and, therefore, into the heart muscle. It is also possible that MSC EVs that enter the myocardium slow down the elimination of toxic, and antigenic substances that have fallen heart with the blood from PCT, also due to immunomodulatory effect. However, gradually, the action of MSC EVs stops, and the activity of the inflammatory process is restored. Immunocompetent cells begin lysis of debris, which, if it is impossible to eliminate outward (consolidation of the edges of the surgical incision) [18], enters the blood in a larger volume and is disseminated throughout the body, ending in the myocardium. Leukocytes migrate to places with a high concentration of this detritus, and since debris was in the blood and tissues of the heart later, the number of immunocompetent cells increases in the myocardium also with a delay and remains elevated for a longer period.

In other words, only a very small part of MSC EVs reaches the myocardial capillaries and the EV effect on the heart, including the number of tissue leukocytes, is insignificant. It is more likely that the found changes are due to the influence of detritus coming from the surgical site. Additionally, since after the use of MSC EVs, which have an immunomodulatory effect, the detritus in the operation place is lysed more slowly, there is more debris in the blood and, accordingly, in the heart; therefore, the adverse effect of this detritus on the myocardium is more pronounced. It is due the need to lysis of this debris in the myocardium that the number of neutrophils and macrophages increases more pronouncedly. Vascular reactions of the myocardium are also mainly due to the presence of detritus and not the influence of MSC EVs. Additionally, due to the large volume of detritus trapped in the myocardium after the use of MSC EVs, the period of elimination of this detritus from the heart increases; in fact, “cleansing”, which is the normalization of the myocardial status, is slower.

Statistically significant differences in vascularization and inflammatory infiltration between the myocardium of the right and left ventricles of the rabbit hearts were not found, both after only damage to PCT and as a result of the creation of a PCT defect with subsequent administration of MSC EVs. On the one hand, a smaller volume of both MSC EVs and detritus, formed during damage to PCT, enters the tissues of the left ventricle with blood flow. On the other hand, MSC EVs and the soluble components of debris from PCT along the blood vessels fall directly into the right ventricle, and after passing through the lungs, they find themselves in the left ventricle, penetrate into the coronary arteries, and spread throughout the whole myocardium, both the left and the right parts of the heart.

In addition, we did not expect such pronounced myocardial reactions; the death of one rabbit was noted on the 5th day after the surgery with the use of MSC EVs. It is unlikely that the death of the animal was due either to a surgical infection (sepsis) or to painful shock during the rabbit’s movements. However, it is possible that this death may be associated with the detected changes in the heart, especially since MSC EVs contribute to a more pronounced myocardial pathology.

## 4. Conclusions

As a result of modeling the PCT defect, rabbits develop myocardial edema in the heart muscle by the 3rd day, the lymphatic vessels expand; then, on the 7th day, the blood vessels become wider. In the myocardium, the relative and absolute contents of neutrophils, erythrocytes, and macrophages increase, but the percentage of lymphocytes decreases. By day 10, almost all of these changes return to their initial values. The detected transformations of the myocardium are most likely due to the ingress of detritus with the blood flow from the PCT. The use of MSC EVs to influence the regeneration of damaged tissue of PCT promotes earlier dilatation of the blood vessels of the heart with pronounced diapedesis of erythrocytes or even hemorrhages, prolongation of edema, the formation of blood clots in vessels with obliteration of their lumen, sclerotic transformation of vascular walls and paravascular tissues. In the myocardium, the number density of neutrophils, and the percentage of lymphocytes and neutrophils become smaller, with a simultaneous increase in the relative numbers of erythrocytes and macrophages. Changes in the content of macrophages remained until the end of the observation—up to 10 days after the surgery. The discovered effect of MSC EVs is most likely associated with the suppression of the activity of the inflammatory process in the PCT area, which, in turn, was caused by a longer ingress of detritus with blood flow into the myocardium. The absence of statistically significant differences between changes in the myocardium of the left and right ventricles may indicate that both detritus from the surgical site and MSC EVs affect the heart spreading through the coronary artery system.

## Figures and Tables

**Table 1:** The structure of the myocardium of the left ventricle in rabbits at different times after damage to the PCT and the introduction of MSC EVs (S ± σ).

Structures	Intact Control	Time after Surgery		
		3 Days	7 Days	10 Days
***Damage of PCT without MSC EVs***				
Vessels blood (A_A_) lymphatic (A_A_)	0.778 ± 0.833 0.889 ± 0.782	1.11 ± 1.17 3.78 ± 1.09 ^#^	4.78 ± 0.833 ^#,$^ 5.56 ± 1.33 ^#^	1.33 ± 1.12 ^&^ 2.56 ± 1.01
Interstitial spaces (A_A_)	0.778 ± 0.833	12 ± 1.66 ^#^	13 ± 1.22 ^#^	2 ± 0.707 ^$,&^
Lymphocytes (%) (N_A_)	79.7 ± 3.57 443 ± 63	63.3 ± 2.29 ^#^ 606 ± 107	53.7 ± 3.61 ^#,$^ 477 ± 56.8	57.7 ± 4.53 ^#^ 532 ± 78.9
Neutrophils (%) (N_A_)	1.89 ± 0.782 10.3 ± 3.97	7.67 ± 1.32 ^#^ 72.8 ± 16.5 ^#^	7.78 ± 0.833 ^#^ 69.2 ± 10.9 ^#^	4 ± 0.707 ^#,$,&^ 37 ± 8.86 ^#,&^
Erythrocytes (%) (N_A_)	1.56 ± 0.726 8.78 ± 5.07	3.78 ± 0.667 ^#^ 36.3 ± 9.23 ^#^	3.56 ± 0.527 ^#^ 31.6 ± 5.53 ^#^	2 ± 0.866 17.9 ± 7.06
Macrophages (%) (N_A_)	12 ± 2.55 66.3 ± 14.4	13.7 ± 1.32 130 ± 23.3 ^#^	24.8 ± 2.6 ^#,$^ 220 ± 34.6 ^#,$^	27.7 ± 3.39 ^#,$^ 256 ± 45.5 ^#,$^
***Damage of PCT with MSC EV introduction***				
Vessels blood (A_A_) lymphatic (A_A_)	0.778 ± 0.833 0.889 ± 0.782	4.25 ± 0.754 ^#,^* 6.75 ± 0.965 ^#,^*	3.78 ± 0.667 ^#^ 4.11 ± 0.782 ^#,$^	1 ± 1.12 ^$,&^ 1.89 ± 0.928 ^$^
Interstitial spaces (A_A_)	0.778 ± 0.833	8.17 ± 0.937 ^#,^*	12.3 ± 1.32 ^#,$^	14.4 ± 1.59 ^#,$,^*
Lymphocytes (%) (N_A_)	79.7 ± 3.57 443 ± 63	50.1 ± 3.26 ^#,^* 380 ± 71.2	50.3 ± 3.67 ^#^ 459 ± 62.9	51.4 ± 4.42 ^#^ 550 ± 96.3
Neutrophils (%) (N_A_)	1.89 ± 0.782 10.3 ± 3.97	4.25 ± 0.754 ^#,^* 32.6 ± 9.49 ^#,^*	7.56 ± 1.13 ^#,$^ 69.1 ± 14.8 ^#,$^	7.44 ± 1.01 ^#,$,^* 78.6 ± 7.89 ^#,$,^*
Erythrocytes (%) (N_A_)	1.56 ± 0.726 8.78 ± 5.07	6.5 ± 1.17 ^#,^* 48.9 ± 9.91 ^#^	4 ± 0.866 ^#^ 36.1 ± 7.13 ^#^	1.78 ± 0.833 ^$^ 18.9 ± 9.25 ^$^
Macrophages (%) (N_A_)	12 ± 2.55 66.3 ± 14.4	26.3 ± 2.86 ^#,^* 199 ± 37.4 ^#^	23.9 ± 1.45 ^#^ 217 ± 26.9 ^#^	27.1 ± 3.3 ^#^ 288 ± 42 ^#^

Note: * values significantly differing from the corresponding after surgery without MSC EVs (*p* ≤ 0.05); ^#^ values significantly differing from the corresponding in intact control (*p* ≤ 0.05); ^$^ values significantly differing from the corresponding on the 3rd day after the surgery (*p* ≤ 0.05); ^&^ values significantly differing from the corresponding on the 7th day after the surgery (*p* ≤ 0.05); A_A_ is the relative area of the structures in the section (% from section area); N_A_ is the numerical density of cells per 10^5^ μm^2^ in the section.

## Data Availability

The data presented in this study are available on request from the corresponding author.
